# Does cemented or cementless single-stage exchange arthroplasty of chronic periprosthetic hip infections provide similar infection rates to a two-stage? A systematic review

**DOI:** 10.1186/s12879-016-1869-4

**Published:** 2016-10-10

**Authors:** D. A. George, N. Logoluso, G. Castellini, S. Gianola, S. Scarponi, F. S. Haddad, L. Drago, C. L. Romano

**Affiliations:** 1 Department of Trauma and Orthopaedic Surgery, University College London Hospitals, London, UK; 2 Centre for Reconstructive Surgery and Osteoarticular Infections, Orthopaedic Research Institute Galeazzi, Milan, Italy; 3 Department of Biomedical Sciences for Health, University of Milan, Milan, Italy; 4 IRCCS Galeazzi Orthopaedic Institute, Unit of Clinical Epidemiology, Milan, Italy; 5 Center of Biostatistics for Clinical Epidemiology, School of Medicine and Surgery, University of Milano-Bicocca, Monza, Italy; 6 Clinical Chemistry and Microbiology Lab, IRCCS Galeazzi Institute, Milan, Italy

**Keywords:** Infection, Periprosthetic hip infections, Exchange arthroplasty, Single-stage, Two-stage, Cemented, Cementless

## Abstract

**Background:**

The best surgical modality for treating chronic periprosthetic hip infections remains controversial, with a lack of randomised controlled studies. The aim of this systematic review is to compare the infection recurrence rate after a single-stage versus a two-stage exchange arthroplasty, and the rate of cemented versus cementless single-stage exchange arthroplasty for chronic periprosthetic hip infections.

**Methods:**

We searched for eligible studies published up to December 2015. Full text or abstract in English were reviewed. We included studies reporting the infection recurrence rate as the outcome of interest following single- or two-stage exchange arthroplasty, or both, with a minimum follow-up of 12 months. Two reviewers independently abstracted data and appraised quality assessment.

**Results:**

After study selection, 90 observational studies were included. The majority of studies were focused on a two-stage hip exchange arthroplasty (65 %), 18 % on a single-stage exchange, and only a 17 % were comparative studies. There was no statistically significant difference between a single-stage versus a two-stage exchange in terms of recurrence of infection in controlled studies (pooled odds ratio of 1.37 [95 % CI = 0.68-2.74, I^2^ = 45.5 %]).

Similarly, the recurrence infection rate in cementless versus cemented single-stage hip exchanges failed to demonstrate a significant difference, due to the substantial heterogeneity among the studies.

**Conclusion:**

Despite the methodological limitations and the heterogeneity between single cohorts studies, if we considered only the available controlled studies no superiority was demonstrated between a single- and two-stage exchange at a minimum of 12 months follow-up. The overalapping of confidence intervals related to single-stage cementless and cemented hip exchanges, showed no superiority of either technique.

## Background

There remains an ongoing discrepancy in the literature between the infection recurrence rates after a single-stage exchange arthroplasty compared to a two-stage exchange for chronic periprosthetic hip infections. Infection has been reported as the third reason for revision after total hip arthroplasty in the USA [1], complicating 0.5 to 2 % of primary arthroplasties [2–4].

The operative approach is determined by a combination of surgeon, patient, joint, and infection factors. Literature regarding the optimal inclusion and exclusion criteria for each modality is varied, but there is a general consensus that a two-stage exchange should be undertaken in patients with unknown pathogens or those of high-virulence [5–7].

Previous attempts at addressing this issue have been undertaken by various prospective [8–10] and retrospective cohort studies [11–13] comparing the modalities used, or systematic reviews [14, 15], but due to various limitations, such as determining the ‘ideal candidate’ for each treatment, a definitive conclusion has not been shown.

In the lack of large prospective, randomised controlled comparative trials, this comprehensive systematic review and meta-analysis of observational studies was undertaken to investigate the relative efficacy, in terms of recurrence of the infection, in a single- compared to two-stage exchange arthroplasty for chronic periprosthetic hip infection. A similar review has been recently reported for periprosthetic knee and shoulder infections [16, 17]. In addition, we aim to further analyse the infection rates after cemented and cementless single-stage exchanges, which have not been previously undertaken.

## Methods

### Search startegy

We searched for studies published up to December 2015 on the following databases: EMBASE; PubMed/Medline; Medline Daily Update; Medline In-Process and other non-indexed citations; Google Scholar; SCOPUS; CINAHL; Cochrane Central Register of Controlled Trials and Cochrane Database of Systematic Reviews; NHS Health Technology Assessment; http://www.google.com; and http://www.yahoo.com. The search was executed using MeSH and text keywords [see Appendix 1] and adapted for each database in order to achieve more sensitivity. Original study reports as well as review articles were retrieved, and the reference lists from all reviewed articles were assessed to complete the literature search. No language restrictions were applied.

### Eligibility criteria

We included studies that fuflilled the following inclusion criteria: (a) sample of at least 4 patients with prosthetic hip infection that underwent a surgical revision; (b) single-stage or two-stage exchange arthroplasty as surgical treatment; (c) a minimum follow-up of 12 months; (d) study reporting results relating to delayed or chronic infection (6 weeks or later) stages of disease; (e) recurrent infection after treatment as outcome; (f) study design classifiable as comparative study, prospective or retrospective study with no compared group.

### Study selection

Two investigators independently searched and reviewed the literature and classified the references in terms of whether they should be included on the basis of the title and abstract. In order to include all studies, if full text was not avaiable, abstracts with enough information to be qualitative and quantitative assessed were included. If more than one paper by the same author(s) was retrieved and their follow-ups were found to overlap, only the most recent reference with the longest follow-up and largest patient series was included. Discrepancies were solved by consusus.

### Data extraction

Data collection was performed by four reviewers. The following data were extracted: name of author, year of publication, type of study design, minimum, maximum and mean period of follow up, number of patients included and number of recurrent infections (in case of comparative studies number of patients per group).

### Outcome

Our primary outcome was the recurrent infection rate. We chose to extract data only of patients who completed the single-stage or the two-stage revision. We did not include patients that had received a supplemental revision for a new infection following the prior septic revision, nor those who did not receive the complete reimplantation process, or died for cause unrelated to infection recurrence.

### Quality assessment

In order to reflect the information expected to be present in each included study, as a measure of quality we selected and evaluated the following two bias: (1) retrospective or prospective analysis and source of data (record bias); (2) relevance and definition of measured outcome for infection (reporting bias). Two independent reviewers performed the quality assessment; disagreements were resolved by consensus.

### Statistical analysis

Infection recurrence rates were treated as dichotomous variables using the odds ratio (OR) for meta-analysis of controlled studies (single-stage versus two-stage) and the ratio between number of infection and total number of patients for proportional meta-analysis of cohort studies reporting only one treatment group, along with 95 % confidential intervals (CI).

The analysis was performed using extracted patient data from the individual studies. Because of the differences among the included studies and several uncontrolled variables, we used a random-effect model [18]. The results from individual trials were combined when possible, but otherwise single forest plots will be reported without the overall duration of follow-up.

In single forest plot, each horizontal line on the graph represents a case series included in the meta-analysis. The estimated effect is marked with a solid black square, and the size of the square represents the weight of the corresponding study plotted in the meta-analysis. The combined total estimate is marked with an unfilled diamond at the bottom of the forest plot. Statistical heterogeneity was assessed using the I^2^ statistic and assume influential when the I^2^ was greater than 50 % and *p* < 0.05 as statistically significant for the calculation of heterogeneity; I^2^ illustrates the percentage of the variability in effect estimates resulting from clinical and/or methodological heterogeneity rather than sampling error [19, 20].

Forest plots were presented for the following interventions: single-stage, two-stage, single-stage cemented, and single-stage cementless hip arthroplasties. The presence of an overlap of the confidence intervals from the two interventions, for example between single-stage and two stage exchanges, suggests similar effect of the interventions on the outcome. Alternatively, non-overlapping CIs suggest different effects from the interventions studied [21].

We used the following software: StatsDirect [StatsDirect Ltd, Cheshire, UK] for the proportional meta-analyses and Review Manager [RevMan version 5.2, The Cochrane Collaboration, The Nordic Cochrane Centre, Copenhagen 2012] for meta-analyses in controlled studies.

## Results

### Selection and characteristics of studies

**Fig. 1:**
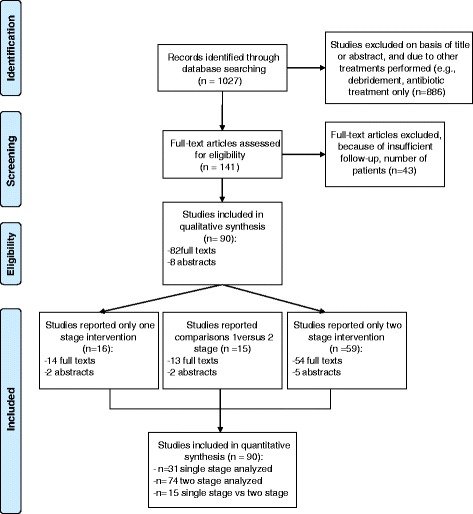
Flow diagram of study selection process

The results of the study selection are shown in Fig. 1. We found 90 original observational studies. Sixteen studies reported the results only after a single-stage exchange, 59 reported only a two-stage hip exchange and 15 reported the comparison of a single-stage versus a two-stage. Overall, 31 original studies reported data about single-stage hip exchange arthroplasty (number of patients, *n* = 1608), which included 27 full text and 4 abstracts. Seventyfive studies reported on two-stage exchanges (*n* = 3679), of which 68 were full texts and 7 abstracts. Characteristics of the included studies are summarized in Table 1.

**Table 1:** General characteristics, record bias and reporting bias of included studies

Study First Author	Ref.	Year	Patients (*n*)	Stage Investigated	Follow Up (months)			Design	Record Bias	Reporting Bias (Outcome Measure)
					Min	Max	Mean			
Babiak	[28]	2012	9	Two	36	180	84	NA	Yes	NA
Babis	[29]	2015	31	Two	20	48	30	Retrospective	Yes	Symptoms, Imaging, Laboratory
Berend	[30]	2013	186	Two	24	180	53	Retrospective	NR	Culture
Biring	[31]	2009	48	Two	120	180	144	Retrospective	Yes	Culture
Bori	[32]	2014	24	Single	25	94	45	Retrospective	Yes	Culture
Buchholz	[33]	1981	583	Single	24	132	–	Prospective	Yes	Culture
Buttaro	[34]	2005	29	Two	24	60	32.4	Retrospective	Yes	Symptoms, Imaging, Laboratory
Cabrita	[8]	2007	38	Two	24	102	48	Prospective	Yes	Culture
Callaghan	[35]	1999	12	Single	12	168	109.2	Retrospective	Yes	Imaging
Camurcu	[36]	2015	41	Two	24	96	54	Retrospective	Yes	Culture
Carlsson	[37]	1985	72	Both	12	72	–	Prospective	Yes	Symptoms, Imaging, Culture
Chen	[38]	2015	155	Two	36	180	116.4	Retrospective	NR	Culture
Choi	[12]	2013	61	Both	12	132	61	Retrospective	Yes	Culture
Colyer	[10]	1994	37	Two	12	88	36	Unclear	Yes	Culture
Cordero-Ampuero	[39]	2009	36	Two	12	144	52.8	Prospective	Yes	Culture
D'Angelo	[40]	2011	28	Two	18	106	53	Retrospective	Yes	Symptoms, Imaging, Laboratory
Darley	[41]	2009	19	Two	24	36	26	Prospective	Yes	Symptoms, Culture
De Man	[42]	2011	72	Both	17	204	60	Retrospective	Yes	Culture
Degen	[43]	2012	30	Two	24	70	43	Retrospective	Yes	Symptoms, Culture
Ekpo	[44]	2014	19	Two	24	132	48	Retrospective	Yes	Laboratory, Culture
Evans	[45]	2004	23	Two	24	108	48	Prospective	NR	Symptoms, Culture
Fehring	[46]	1999	25	Two	24	98	41	Prospective	Yes	Symptoms, Laboratory
Fink	[47]	2009	36	Two	24	60	35	Prospective	Yes	Culture
Fitzgerald	[48]	1985	131	Two	24	108	49	Retrospective	Yes	Symptoms
Gao	[49]	2008	15	Both	12	37	19	NA	NA	Culture
Garvin	[50]	1994	40	Both	24	120	60	NA	NA	Culture
Haddad	[51]	2000	50	Two	24	104	69.6	Retrospective	Yes	Symptoms
Hofmann	[52]	2005	27	Two	28	148	76	Retrospective	Yes	Symptoms, Imaging, Laboratory
Hope	[53]	1989	80	Both	2	121	–	Retrospective	Yes	Culture
Hsieh	[55]	2004	128	Two	24	96	58.8	Retrospective	Yes	Symptoms, Laboratory
Hsieh	[11]	2009	99	Two	24	60	43	Retrospective	Yes	Symptoms, Culture
Hsieh	[54]	2013	28	Two	48	120	86	Retrospective	Yes	Symptoms,Culture, Laboratory
Hughes	[24]	1979	26	Both	32	83	51	Retrospective	Yes	Symptoms, Imaging, Laboratory, Culture
Ibrahim	[56]	2014	125	Two	60	75	103.2	Retrospective	Yes	Symptoms, Laboratory, Culture
Ilchmann	[57]	2015	38	Single	24	181.2	79.2	Retrospective	Yes	Symptoms, Culture
Jenny	[58]	2014	63	Single	36	72	–	Retrospective	Yes	Symptoms, Culture
Johnson	[59]	2013	66	Two	24	105	45	Retrospective	Yes	Symptoms, Laboratory, Culture
Karpas	[60]	2003	18	Two	24	120	42	Retrospective	Yes	NR
Kent	[61]	2010	12	Two	26	60	38	Retrospective	NR	NR
Ketterl	[13]	1988	161	Two	24	168	32	NA	NA	NA
Kim	[63]	2011	130	Two	60	168	124.8	Retrospective	Yes	Laboratory, Culture
Klouche	[22]	2012	84	Both	24	68	35	Prospective	Yes	Culture
Koo	[62]	2001	22	Two	24	78	41	Prospective	Yes	Symptoms, Imaging, Laboratory
Lai	[64]	1996	39	Two	30	84	48	Prospective	Yes	Symptoms, Laboratory
Lee	[65]	2013	17	Two	24	96	48	Retrospective	Yes	Symptoms, Laboratory, Culture
Leung	[66]	2011	38	Two	24	123	58	Retrospective	Yes	Symptoms,Laboratory
Li	[67]	2015	10	Both	78	187.2	103.2	Retrospective	Yes	Symptoms, Laboratory, Culture
Lieberman	[68]	1994	32	Two	24	74	40	Retrospective	NR	NR
Macheras	[69]	2012	35	Two	84	168	139.2	Retrospective	Yes	NR
Magnan	[70]	2001	8	Two	24	48	35	Retrospective	NR	NR
Masri	[71]	2007	29	Two	24	88	47	Retrospective	Yes	Symptoms, Laboratory
McDonald	[72]	1989	81	Two	24	163.2	66	Prospective	Yes	Culture
McKenna	[73]	2009	30	Two	24	60	35	Retrospective	Yes	Laboratory
Miley	[74]	1982	46	Single	32	–	48.5	Prospective	NR	Unclear
Morales	[75]	1999	37	Two	36	156	57.6	NA	NA	NA
Morscher	[76]	1994	74	Both	12	132	84	NA	Yes	Symptoms, Imaging, Laboratory
Mulcahy	[77]	1996	15	Single	24	84	53	Retrospective	Yes	Symptoms, Imaging, Laboratory, Culture
Nestor	[78]	1994	34	Two	24	72	47	Retrospective	Yes	Culture
Neumann	[79]	2011	44	Two	36	120	67	Retrospective	Yes	Laboratory
Nusem	[80]	2006	18	Two	60	168	108	Retrospective	Yes	Unclear
Oussedik	[9]	2010	50	Both	66	105.7	81.6	Prospective	Yes	Imaging, Laboratory
Pignatti	[81]	2010	41	Two	60	120	63.6	Retrospective	Yes	Symptoms, Imaging, Laboratory
Raut	[82]	1995	57	Single	24	151	88	Prospective	Yes	Symptoms, Laboratory
Romanò	[83]	2012	183	Two	24	104	56	Retrospective	Yes	Laboratory, Culture
Rudelli	[84]	2008	32	Single	24	96	52.8	Unclear	Yes	Imaging, Laboratory, Culture
Sabry	[85]	2013	78	Two	24.3	135.3	58	Retrospective	Yes	Symptoms, Laboratory
Sanchez	[86]	2009	168	Two	24	192	84	Retrospective	Yes	Symptoms, Culture
Sanzen	[87]	1988	102	Both	24	108	–	Prospective	Yes	Culture
Schneider	[88]	1989	26	Single	12	108	–	NA	Yes	NA
Schwarzkopf	[89]	2014	56	Two	12	–	32.4	Retrospective	Yes	Laboratory, Culture
Seung-Jae	[90]	2009	34	Two	24	120	52.8	Retrospective	Yes	Symptoms, Culture
Stockley	[91]	2008	114	Two	24	175	74	Prospective	Yes	Symptoms, Laboratory, Culture
Sudo	[25]	2008	7	Two	27.6	73.2	60	Retrospective	Yes	Symptoms, Imaging, Laboratory
Takigami	[92]	2010	8	Two	24	81	49	Retrospective	Yes	Symptoms, Laboratory
Thabe	[93]	2007	16	Two	72	120	75.6	Prospective	Yes	NR
Toulson	[94]	2009	82	Two	24	203	64.8	Retrospective	Yes	Unclear
Ure	[95]	1998	20	Single	42	205.2	118.8	Prospective	Yes	Symptoms, Imaging, Laboratory, Culture
van Diemen	[96]	2013	136	Two	24	180	72	Retrospective	Yes	Symptoms, Imaging, Laboratory, Culture
Wang	[97]	2011	12	Two	36	96	64.8	NA	Yes	NR
Weber	[98]	1986	33	Both	60	96	72	Retrospective	NR	Laboratory
Whittaker	[99]	2009	41	Two	25	83	49	Retrospective	Yes	Culture, Laboratory
Wilson	[100]	1974	19	Single	24	–	–	Prospective		Culture
Wilson	[24]	1989	22	Both	36	120	60.2	Prospective	Yes	Symptoms, Imaging, Laboratory
Winkler	[27]	2008	37	Single	63	183	103	Prospective	Yes	Symptoms, Imaging, Laboratory
Wolf	[101]	2014	92	Both	24	–	–	Retrospective	Yes	Symptoms, Laboratory, Culture
Wroblewski	[23]	1986	101	Single	38.8	–	–	Prospective	NR	NR
Yamamoto	[102]	2003	17	Two	14	62	38	Retrospective	Yes	Laboratory
Yoo	[103]	2009	12	Single	39.6	135.6	86.4	Prospective	Yes	Laboratory, Culture
Younger	[104]	1997	48	Two	24	63	43	Prospective	Yes	Culture
Zeller	[105]	2014	99	Single	24	–	41.6	Prospective	Yes	NA

The number of patients undergoing a single-stage exchange ranged from 12 to 583, with a follow-up of 12 to 183 months. Considering a single-stage exchange performed with *cementless* implants (with or without antibiotic-loaded bone grafts) we found a total of 148 patients (mean follow-up: 78.1 months) whereas for single-stage exchange performed with *cemented* implant involved 1271 patients (mean follow-up: 78.1 months). The number of cases for only two-stage exchange studies ranged from 7 to 186, with a follow-up of 12 to 203 months.

### Quality assessment

The quality of included studies is shown in Table 1. Overall, 62 % of included studies were retrospective, 29 % prospective and 8 % were not definable because the full text was unavailable. Observational studies can produce high quality information but, given the nature of these study design, the lack of a control group and the likely confounding variables, the methodological quality was limited leading to difficult generalisation of results. The outcome was specified in the majority of the studies (84 %), selecting infection recurrence as the elective outcome to reflect the success of the two types of interventions. In the half of the included studies, the infection recurrence was diagnosed with more than two measurements (i.e. positive culture, clinical symptoms, imaging etc.). Nevertheless, a unique and universal definition of ‘hip periprosthetic infection’ was not adopted and among studies.

Concerning data reporting, only 57.3 % of the studies gave a description of their criteria for selecting either a single- or two-stage exchange arthroplasty. Other relevant variables such as the indication for primary hip arthroplasty or host type were poorly reported (49.0 % and 36.5 % respectively). Other variables, such as age (90.7 %), gender (86.4 %), isolated pathogen (91.6 %), duration of interim period between stages (88.6 %), implant type used at exchange arthroplasty (72 %), length of antibiotic therapy (76.3 %), number of patients lost to follow-up (73.8 %) were more often reported.

### Recurrent infection

#### Single-stage vs two-stage

We have analysed the data using a random-effects model to incorporate the wide range of variables.

**Fig. 2:**
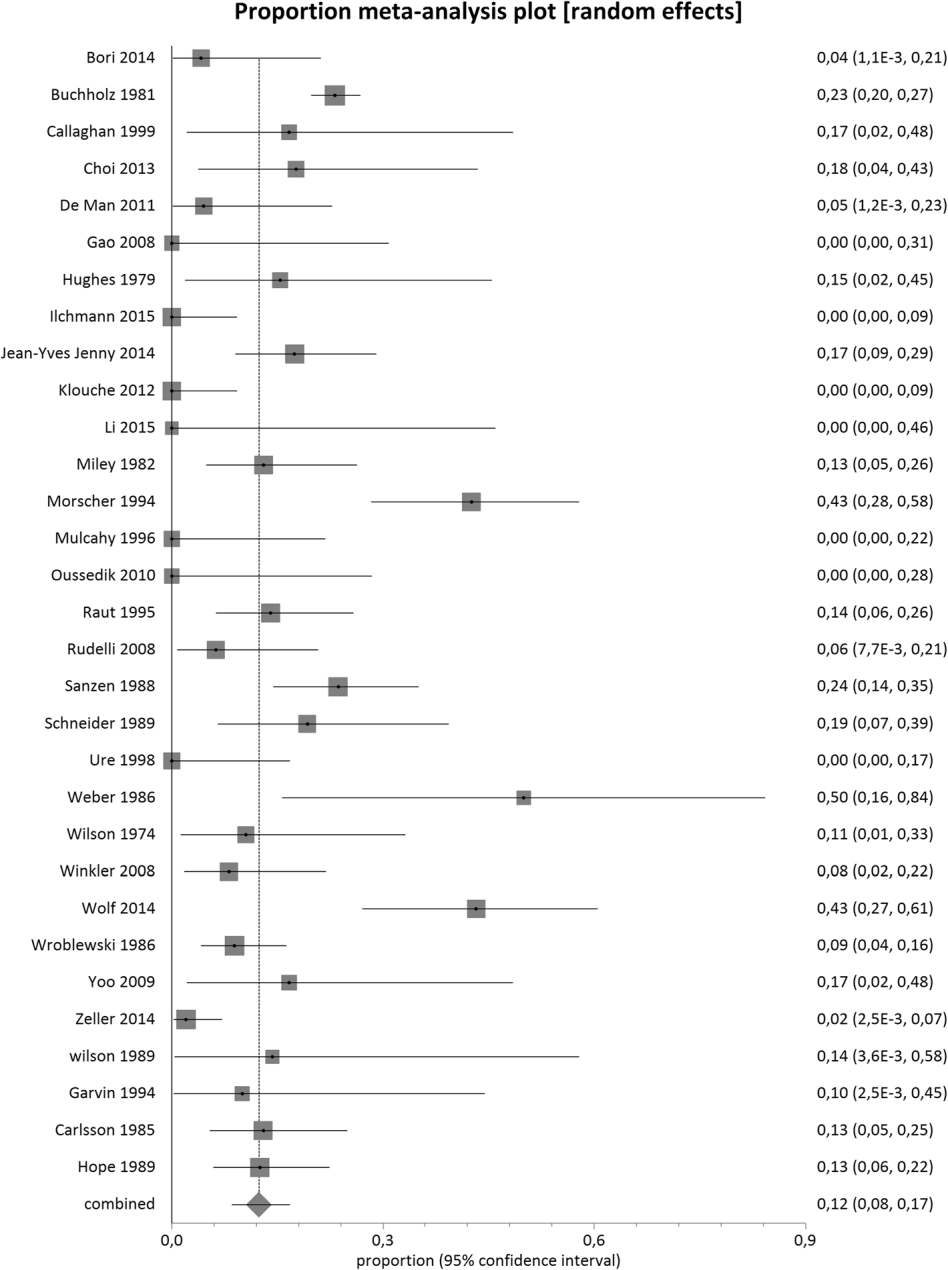
Proportional meta-analysis regarding infection recurrence after single-stage hip arthroplasty

The mean pooled proportion of recurrent infection was 12 % (95 % CI = 8 %-17 %) in single-stage hip exchange (1608 cases, *n* = 31 studies) and demonstrated high clinical and methodological inconsistency between the studies included (I^2^ value = 80.3 %, *p* < 0.0001) (Fig. 2).

**Fig. 3:**
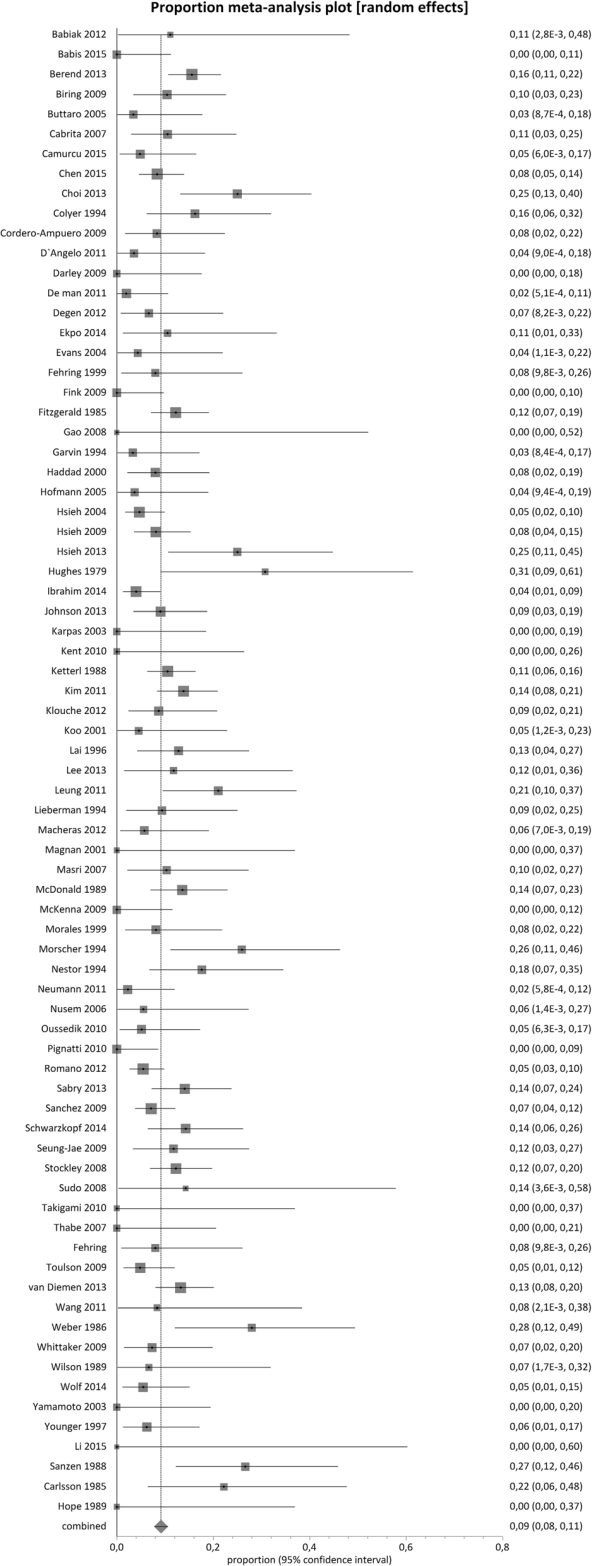
Proportional meta-analysis regarding infection recurrence after two-stage hip arthroplasty

The mean pooled proportion of recurrence of infection was 9 % (95 % CI = 8 %-11 %) in two-stage hip exchange (3679 cases, *n* = 74 studies) and demonstrated moderate clinical and methodological inconsistency between the studies included (I^2^ value = 50.3 %, *p* < 0.0001) (Fig. 3).

**Fig. 4:**
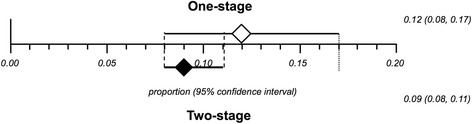
Combined overlapped CIs from single- and two-stage exchange proportional meta-analyses

**Fig. 5:**
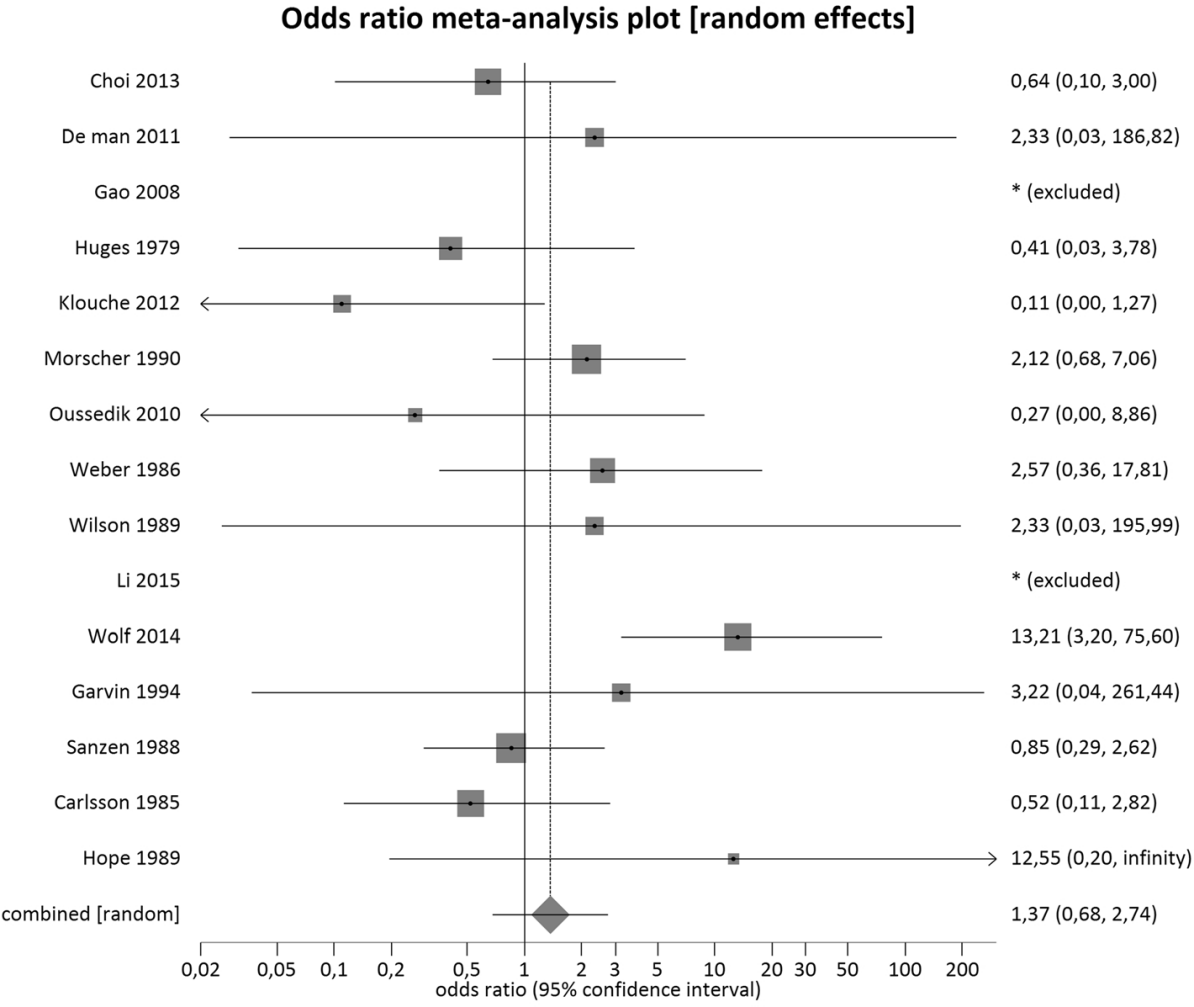
Meta-analysis regarding infection recurrence after single-stage versus two-stage–exchange

The combined overlapped CIs from single- and two-stage exchanges suggests similar effect between the interventions, as represented in Fig. 4. This estimate was confirmed by the comparisons of the available controlled studies (*n* = 15): no statistically significant difference between people undertaking a single- versus a two-stage exchange in terms of recurrence of infection with a pooled odds ratio of 1.37 (95 % CI = 0.68-2.74, I^2^ = 45.5 %, *p* = 0.03) (Fig. 5).

#### Single-stage cementless vs single-stage cemented

The mean pooled proportion of infection recurrence in a single-stage hip cementless exchange (148 cases, n = 6 studies) was 14 % (95 % CI 4 %-28 %), whereas in a cemented exchange (1271 cases, *n* = 19 studies) it was 12 % (95 % CI 7 %-17 %). In both analyses a high clinical and methodological inconsistency was shown between the included studies (I^2^ value = 77.4 % for cementless and I^2^ value = 83.3 % for cemented; *p* < 0.0001).

**Fig. 6:**
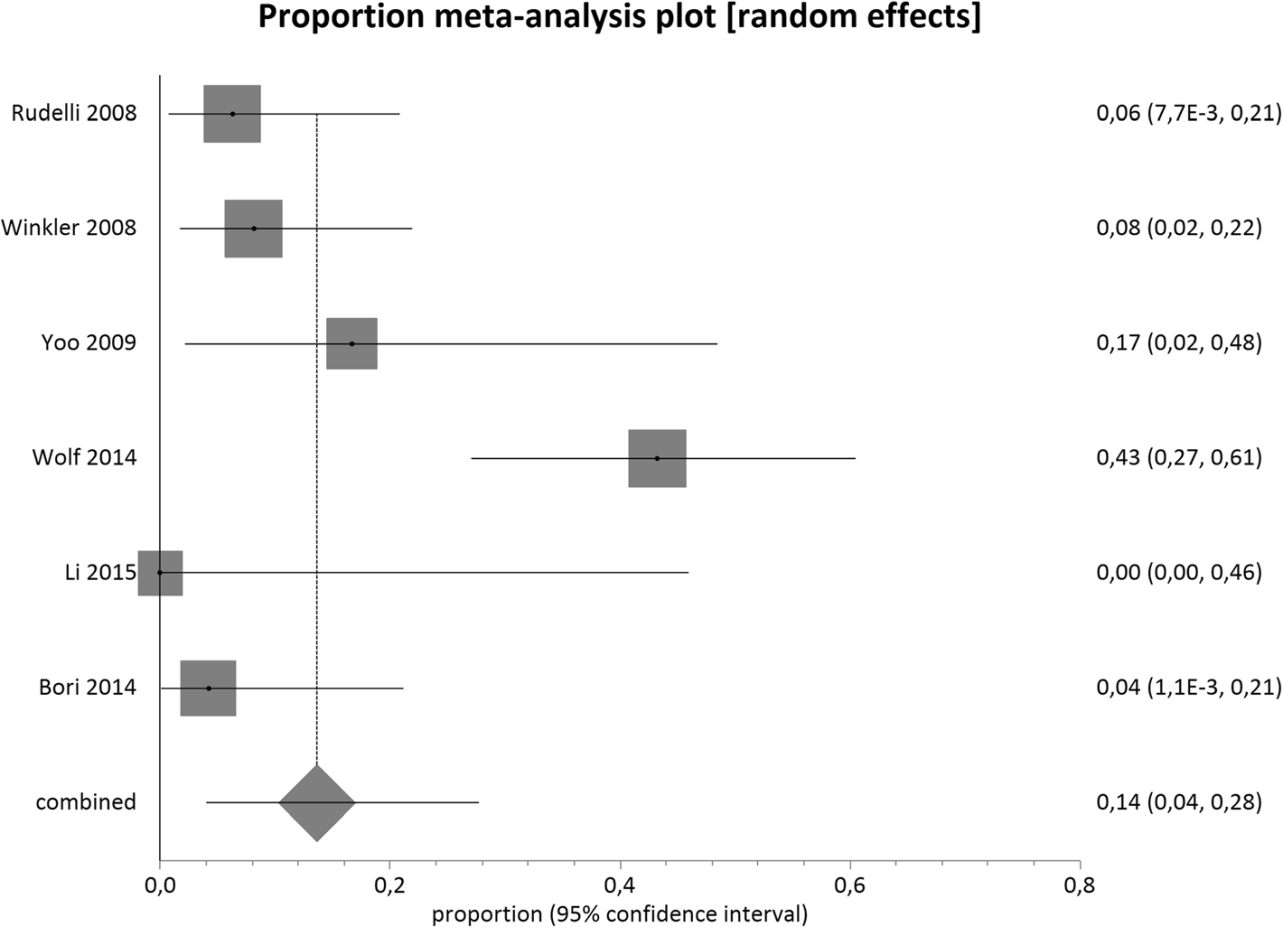
Proportional meta-analysis regarding infection recurrence after cementless single-stage exchange

**Fig. 7:**
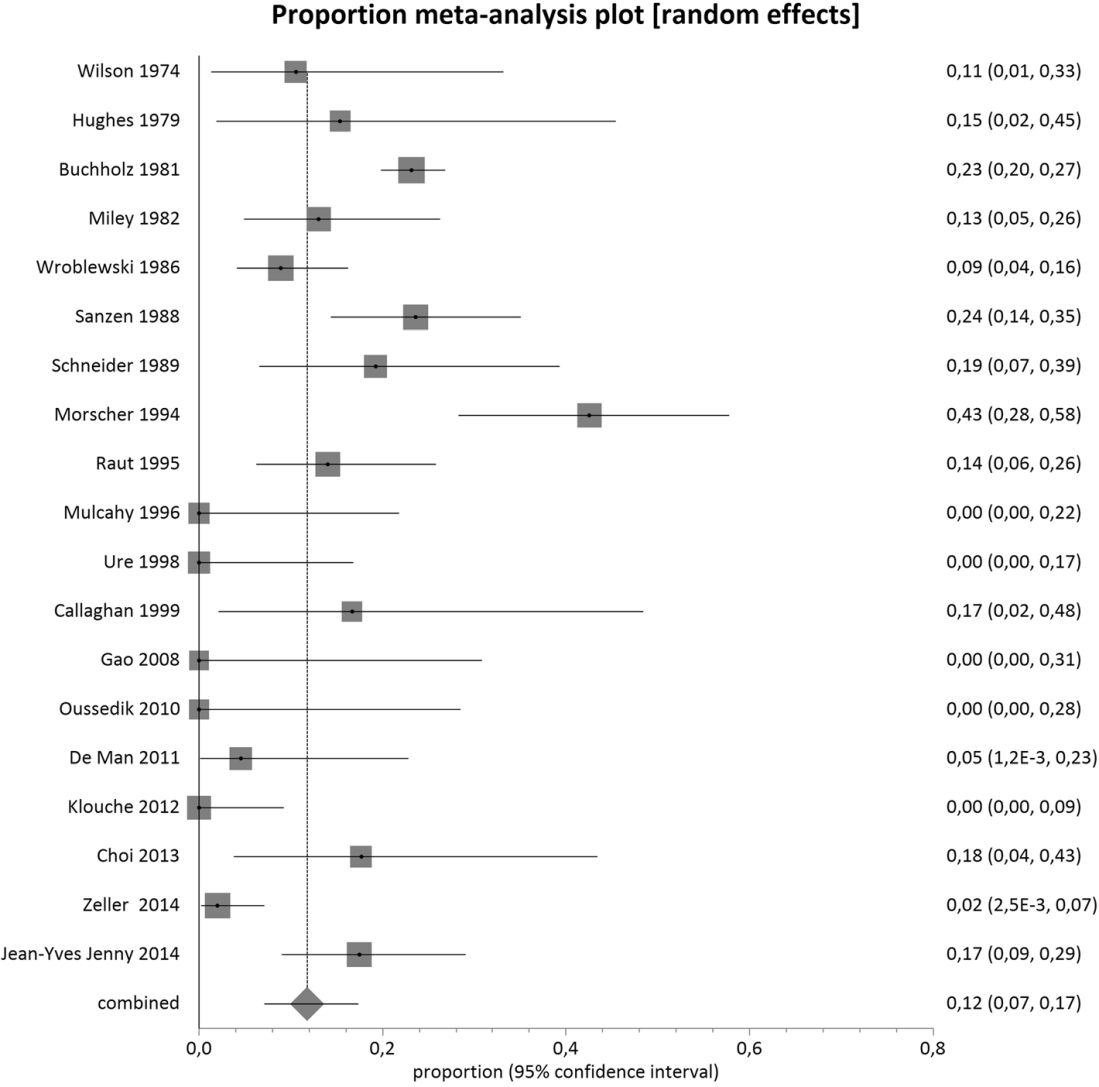
Proportional meta-analysis regarding infection recurrence after cemented single-stage exchange

**Fig. 8:**
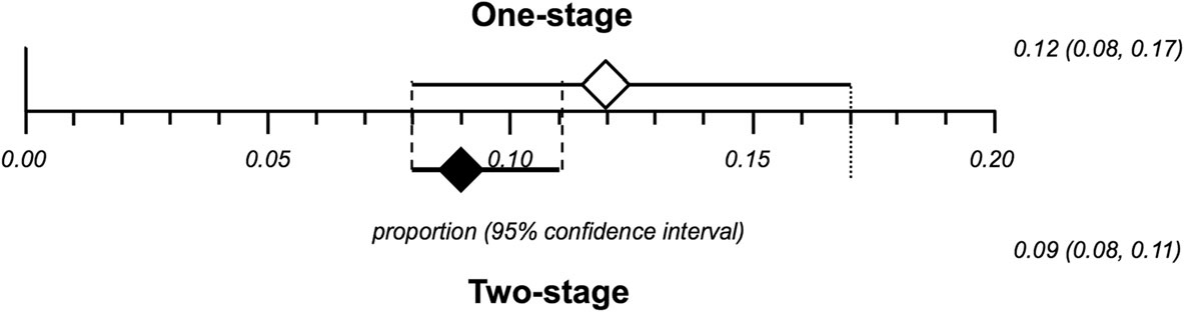
Combined overlapped CIs from cementless and cemented single-stage exchange

Figures 6 and 7 present the pooled proportion for cementless and cemented hip exchanges. The combined overlapped 95 % CIs from cementless and cemented single-stage exchanges suggests similar effect between the interventions studied, as represented in Fig. 8.

## Discussion

This systematic review analyses the current published literature regarding a single- and two-staged exchange for hip periprosthetic infections, where the number of reported two-stage exchange arthroplasty studies largely exceeds that of a single-stage ones.

This study includes a much higher number of studies and patients compared to previous systematic reviews comparing both treatment options in a more limited population [14, 15] and is also, to our knowledge, the first attempt to investigate separately cemented and cementless one-stage revision procedures.

Our results failed to demonstrate a statistical difference between a single- and two-stage exchange arthroplasty, when applying a random effect model. Lange et al. [14] identified only a limited superiority of two-stage exchange arthroplasty in infection eradication, highlighting the low quality of available material, while Beswisk et al. [15] could not demonstrate any difference in eradication rates following a systematic review of studies with a minimum of 24 months of follow-up.

In line with these findings, when considering comparative studies only, the available material did not allow us to prove the superiority of single- or two-stage exchange arthroplasty, while a high heterogeneity of results was observed. As an example, Klouche and co-workers [22], recently reported no infection recurrence after single-stage exchange arthroplasty, even without using antibiotic-loaded cement, while Wolf et al. [23] demonstrated a 43 % infection recurrence rate after single-stage exchange, compared to only 4 % after two-stage revision. Further analysing their data, these authors provided evidence that the difference between the two treatments could be due to the better results obtained with a two-stage approach in more compromised hosts, while either seem to perform equally well, when normal hosts and early infections are involved [23].

Based upon the random-effects model used in our study, the rate of infection recurrence following a single-stage *cementless* exchange arthroplasty is not significantly different from single-stage *cemented* exchange. Once again, the limited number of studies and heterogeneity between both types, cemented and cementless, are worth considering.

More generally, the following limitations of the present study do apply. Patient selection and the eligibility for a single- or two-stage exchange arthroplasty may differ across centers; pathogen and host’s type, implant model and degree of bone loss, type of hip spacer, use and dose of local antibiotics, time interval between stages, post-operative systemic antibiotic treatment, definition of infection, diagnosis and surveillance protocols are all important variables [5, 11, 24–27] that were not reported uniformly across studies and were not considered in the present analysis.

A further limitation of this review concerns the study end-point, that we restricted to reporting infection recurrence, which limits the ability to catch differences in functional outcome, quality of life, or economical impact related to a given surgical option. In addition, we paid attention to the definition of measurements for *recurrence of infection* in order to investigate the “outcome reporting bias” but we were unable to distinguish between recurrent and new infections, as such a distinction was not made in the majority of the studies. The conventional definition of a ‘new’ infection is the isolation of a new microorganism, as opposed to the detection of the same pathogen in ‘recurrent’ infections, however we feel such a differentiation is unreliable. The microbiological results following periprosthetic samples are too unpredictable, especially after previous antibiotic treatment. The criteria for differentiating between recurrent and new infections is weakly supported in the literature, and somewhat artificial [16].

Classifing the design of included studies in order to judge their quality and internal validity was difficult. In fact, for an important part of studies the design assigned was unclear, and considering the inclusion of a paper or abstract published only in English we had an additional limit.

We found a substantial presence of the “record bias” for the majority of studies. Out of the 90 studies included, only 15 studies had a controlled group. The lack of a control group, and the prospective collection of data according to a protocol established before the beginning of the study, can affect the methodological quality limiting the external validity of findings.

We call for the need of large, multi-center randomised controlled trials with higher quality assessment in order to establish the superiority of one type of surgical treatment over another. However, certain circumstantial limitations such as the low incidence of the disease, relatively small patient cohorts, need for long-term follow-up, and variations in microorganisms and patients’ co-morbidities, would also make a large controlled prospective study in this field extremely challenging.

## Conclusion

No superiority was seen for a two-stage exchange arthroplasty over that of a single-stage for chronic periprosthetic hip infections, nor a statistical difference between cemented and cementless single-stage exchanges. This may reflect the shear complexity of this patient cohort and the difficulty in finding the true answer, and further reiterates that the ultimate choice of treatment modality depends on a variety of parameters not addressed in this review. This should include the patient’s preoperative clinical status, potential benefits in function and quality of life to be gained from treatment, its economical implications, and complication rates.

## Appendix 1

Keywords entered either alone or in a variety of combinations during the systematic review process.

Hip: Infection
Arthroplasty: Prosthesis
Total hip replacement: THR
Prosthetic hip infection: Periprosthetic hip infection
Exchange arthroplasty: One-stage
Single-stage: Two-stagerevision

## Abbreviations

CI: Confidential intervals
n: number of patients
OR: Odds ratio
